# Complete genome sequence of *Bacillus pumilus* F12-21, a halotolerant bacterium with antibacterial properties isolated from a Big Bone Lick State Park salt spring

**DOI:** 10.1128/mra.00911-23

**Published:** 2024-03-19

**Authors:** Gretchen Kirchner, Jance Carter, David S. Treves

**Affiliations:** 1 Biology Program, Indiana University Southeast, New Albany, Indiana, USA; SUNY College of Environmental Science and Forestry, Syracuse, New York, USA

**Keywords:** *Bacillus pumilus*, secondary metabolite, antibacterial, salt spring, halotolerant

## Abstract

*Bacillus pumilus* F12-21 is a halotolerant bacterium isolated from a sulfur-enriched salt spring. F12-21 inhibits bacteria of human health interest and bacterial salt spring co-inhabitants. We report the genome of *Bacillus pumilus* F12-21, with a predicted genome of 3.77 Mbp containing 3,732 protein-coding genes, 80 tRNAs, and 24 rRNAs.

## ANNOUNCEMENT

**Fig 1:**
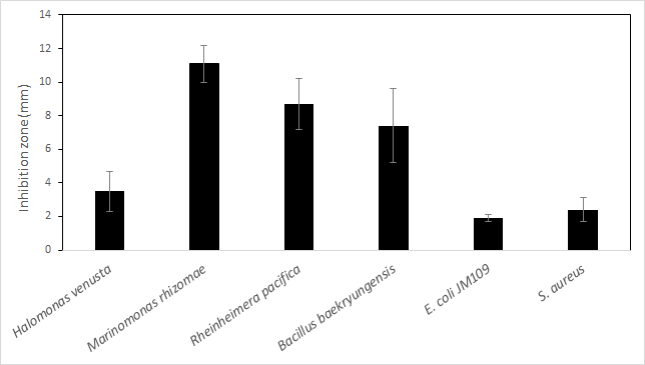
Antimicrobial activity of *Bacillus pumilus* F12-21 toward salt spring co-inhabitants, *E. coli* JM109 and *Staphylococcus aureus*. A spot-on-lawn assay was conducted by spreading 10^8^ CFU of each test strain onto Marine Agar followed by spotting an equal amount of F12-21 in the center of the plates. All stocks were grown in Marine Broth for 24 h at 37°C before plating. After 48 h incubation at 37°C, the clear zone between F12-21 and each test strain was measured. Values are mean ± SEM, *n* = 5. Salt spring isolates were identified by 16S rDNA sequencing (3).

Bacteria produce a wide range of secondary metabolites for defense and communication, and these compounds can be a rich source of new antimicrobial agents (1, 2). While characterizing halotolerant bacteria from Big Bone Lick State Park salt springs (38.8870069 N 84.7507804 W), we identified *Bacillus pumilus* F12-21 that inhibited growth of both co-inhabiting salt spring bacteria and bacteria associated with human and animal infection (Fig. 1). Genome sequencing of *Bacillus pumilus* F12-21 was initiated to investigate this growth inhibition phenotype.

Salt spring sampling was described previously (3). F12-21 was isolated in October 2012 from a salt spring sample incubated on Marine Agar for 48 h at 28°C. F12-21 was purified by subculture on Marine Agar, grown overnight in Marine Broth, and stored in 15% glycerol at −80°C.

F12-21 genomic DNA was isolated from an overnight Luria-Bertani broth culture using the Monarch Genomic DNA Purification Kit (New England Biolabs, Ipswich, MA). Genomic DNA was quantified by Nanodrop and Qubit, and sample integrity was checked using Agilent TapeStation. Genomic DNA was sheared (target size 7–12 kb) with the Megaruptor 3 system (Dioagenode, Denville, NJ), and following slow annealing, PacBio SMRTbell libraries were constructed using the SMRTbell Express Template Prep Kit 2.0 (PacBio, Menlo Park, CA). The pooled library was bound to polymerase using the Sequel Binding Kit 3.0 (PacBio) and loaded onto PacBio Sequel I using the Sequel Sequencing Kit 3.0. Sequencing used one PacBio Sequel SMRT cell resulting in 697,653 polymerase reads and 2,949,305 subreads. The mean subread length was 5,178 bp with an estimated sequencing coverage of 3,636-fold.

Genome assembly was performed by Azenta/Genewiz (South Plainfield, NJ) using SMRT link software v5.1.0 (https://www.pacb.com/support/software-downloads/). Random down-sampling of subreads was set to 200-fold, and *de novo* assembly used Canu v1.9 (4) with coverage cutoff set to 40×. The draft assembly (two contigs, length = 3,796,647 bp) was further refined to merge contigs and remove potential artifacts and then polished with raw subreads using Arrow v2.2.2 (5). Default parameters were used for all software unless otherwise stated. Final genome assembly for *Bacillus pumilus* F12-21 resulted in one contig with a length of 3,769,799 bp and a GC content of 42%.

The assembled genome of *Bacillus pumilus* F12-21 was annotated using the NCBI Prokaryotic Genome Annotation Pipeline v6.5 (6). A total of 3,732 protein-coding genes were identified along with 80 tRNAs, 24 rRNAs, and 3 CRISPR arrays. Several promising biosynthetic gene clusters were identified with AntiSMASH (7), including those coding for lichenysin, bacilysin, and bacillibactin, that may offer insight into the observed antibacterial phenotype. Availability of the *Bacillus pumilus* F12-21 genome sequence will allow further investigation into the antibacterial properties of this bacterium.

## Data Availability

The complete genome of *Bacillus pumilus* F12-21 was deposited in Genbank under accession no. CP132058. The genome sequence described in this report is version CP132058.1. The associated Bioproject and Biosample for F12-21 are PRJNA1001884 and SAMN36823470, respectively. Read data for F12-21 were deposited in the sequence read archive (SRA) under accession number SRX21384642.
